# Firm, Lyft and Relax - A versatile approach for facial rejuvenation: A case series

**DOI:** 10.1016/j.jobcr.2025.05.007

**Published:** 2025-06-03

**Authors:** Marcelo Germani, Ana C.N. Carnevali, Isabela Guerra, Dannyelle L. Rocha, Danielle Dias, Karina Vera Zamorano, Victor R.M. Munoz-Lora

**Affiliations:** a Department of Biological Sciences, Bauru School of Dentistry, University of São Paulo, Brazil; b Federal University of São Paulo, Sao Paulo, Brazil; c Private Office, São Paulo, Brazil; d VeraFondi Clinic, Santiago de Chile, Chile; e Let's HOF Academy, São Paulo, Brazil; f Department of Periodontology and Implantology, University of Guarulhos, Sao Paulo, Brazil

**Keywords:** Aesthetics, Botulinum toxin, Collagen biostimulator, Hyaluronic acid, Stereophotogrammetry

## Abstract

The “Firm, Lyft, and Relax” (FL&R) is a versatile approach that associates botulinum toxin (BTx), hyaluronic acid (HA), and collagen bioestimulators (CBio) treatments, suitable for patients of varying ages and diverse cosmetic concerns. This strategy considers factors such as facial static and dynamic areas, the requirement for projection or volumization, and tissue thickness and firmness. This study aims to elucidate the main concerns of the FL&R technique, presenting a series of four cases and providing a guide for clinicians using combined treatment modalities for facial rejuvenation. The treatments involved the strategic use of BTx, poly-L-lactic acid (PLLA), and HA. Following the treatments, all patients experienced significant aesthetic improvements, as evaluated through 3D stereophotogrammetry. It is important to note that while this approach yields satisfactory outcomes, it should not be standardized; rather, it should be tailored to meet the unique needs of each patient.

## Graphical abstract

Graphical abstract legend:The FL&R approach combines muscle relaxation (Dysport), filling and volumization (Restylane), and collagen stimulation (Sculptra) to address dynamic and static facial areas, projection needs, and tissue thickness, resulting in significant aesthetic improvements, as assessed via 3D stereophotogrammetry.

**Figure:**
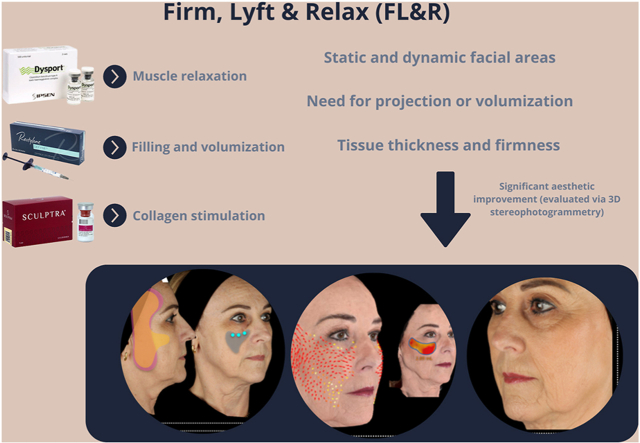

## 1 Introduction

Non-surgical cosmetic treatments have increased in popularity over the past decade, notably driven by the widespread use of hyaluronic acid (HA) and botulinum toxin (BTx) for facial rejuvenation. Together with these primary modalities, collagen biostimulators (CBio) have emerged as a valuable tool aimed at mitigating the effects of aging and enhancing overall facial aesthetics.

Each of these treatments contributes uniquely to the rejuvenation of soft facial tissues. Treatments with HA at strategically chosen anatomical points promote facial tissue projection and repositioning.^1^ Conversely, poly-L-lactic acid (PLLA) enhances skin laxity and dermal thickness, facilitating facial recontouring.^2^ Furthermore, BTx can be employed for tissue repositioning,^3^^,^^4^ as well as improving static and dynamic wrinkles.^5^ While each of these procedures offers significant individual benefits, combining therapies has been demonstrated to yield optimal results, working synergistically for facial rejuvenation.^6^

A recent study introduced the ‘Firm and Lyft’ technique^7^ to delineate a combined therapy employing PLLA (Sculptra, Galderma, Lausanne, Switzerland) along with two specific types of HA gels: Restylane Defyne and Restylane Lyft (Galderma, Lausanne, Switzerland). However, a notable limitation arises from the utilization of only two types of HA and the use of no BTx injections, which may not adequately address the needs of all patients.

For this reason, we propose the Firm, Lyft, and Relax approach (FL&R), a rejuvenation strategy that encompasses treating dynamic wrinkles with BTx, facial restructuring with HA, and enhancing skin laxity with CBio, thereby facilitating soft tissue repositioning and facial rejuvenation. In this study, we present a series of four cases utilizing this structured aesthetic strategy.

## 2 Case series

### 2.1 *The Firm, Lyft and Relax* (FL&R) *strategy*

The FL&R consists of a structured and strategic therapy, including BTx, CBio, and HA administered in a specific sequence.

### 2.2 Botulinum toxin strategy

BTx injections are administered on the upper and lower thirds of the face, with dosage tailored to each patient following individualized assessment. The BTx strategy aims to rebalance the force of mimic muscles by paralyzing depressors, allowing elevator muscles to freely reposition tissues into a cranial position.^3^^,^^4^^,^^8^ To achieve this, a 500-unit Speywood vial of BTx (Dysport, Ipsen, Slough; UK) is reconstituted in 2 ml of sterile saline. Subsequently, BTx is applied using 0.5 ml syringes, with topical anesthesia employed to minimize discomfort from needle insertion.

### 2.3 Collagen biostimulators strategy

CBio is applied laterally to the line of ligaments into the upper, middle, and, if necessary, lower third of the face. This application technique is supported by the concept introduced by Freytag et al. (2022), who emphasize that the ‘line of ligaments' serves as a functional boundary between the lateral and medial facial regions. Applying collagen biostimulators laterally to this line allows for more effective tissue repositioning, utilizing the fascial connections between these areas to facilitate tissue lifting and volumization. This approach minimizes the need for excessive product volume while enhancing the overall facial contour and symmetry.^9^ In these cases, PLLA (Sculptra, Galderma, Brazil) was immediately reconstituted in 8 ml of sterile water and 1 ml of lidocaine, following the manufacturer's instructions.^10^

### 2.4 Hyaluronic acid strategy

Finally, HA injections may be applied to the upper, middle, and/or lower thirds of the face. The selection of the appropriate HA product is guided by its physicochemical characteristics and three critical anatomical variables:

*A. Static versus dynamic areas:* The line of ligaments serves as a reference to delineate between the static and dynamic areas of the face.^11^ The medial region from the line of ligaments is considered dynamic, while the lateral region is deemed static.^12^

Dynamic areas require HA products capable of maintaining natural movement.^1^ Therefore, products with a lower elastic modulus (G′) and greater flexibility are preferred options. Conversely, static areas are better suited for products with lifting capacity, characterized by a higher G'.^9^

B. *Projection versus volumization:* Projection entails a vertical lifting of soft tissues. When projection is required, focal products with a higher G′ are the preferred choice. Conversely, volumization involves horizontal volume gain, and products with a lower G′ and larger particle size are the primary indication.^13^
C. *Tissue thickness:* Facial tissue density plays a critical role in selecting the appropriate product. Tissue thickness assessment is conducted through a detailed physical pinch and observation examination, complemented by the expertise and clinical judgment of the administering professional.^14^ For thinner tissues, products with an intermediate to low G′ are recommended to prevent conspicuousness and ensure a natural appearance. Conversely, for thicker tissues, products with a higher G′ can be employed, providing excellent tissue lifting capability without notable drawbacks.^15^

A complete description of the FL&R approach for each case of this manuscript is shown in Table 1. The products used in this study are commonly utilized by the authors in their clinical practice, based on their proven efficacy. The evaluation of the results was conducted in a standardized manner using 3D stereophotogrammetry to ensure objective and reliable measurements. All procedures followed the ethical standards outlined in the Declaration of Helsinki, and informed consent was obtained from all participants before their data were included in the study.

**Table 1:** Description of Firm, lyft and relax treatment protocol for case 1, 2, 3 and 4.

*Case*	*Treatment*	*Vials/syringe/units*	*Product*	*Area*
Case 1	CBio	2	Sculptra	Pre-auricular/Posterior and anterior temple/Maxilla (First session) Pre-auricular (Second session)
	HA	2	^1^ Restylane Defyne ^1^ Restylane Volyme	Zygomatic Arch/Maxilla
	BTx	24 units Lower Third 36 units Upper Third	Dysport	Frontal/Glabella/Orbicularis/Nasal/Platisma
Case 2	CBio	1	Sculptra	Posterior Temporal/Submalar Region
	HA	3	^2^ Restylane Defyne ^1^ Restylane Volyme	Temple/Mandibular Angle
	BTx	24 units upper third	Dysport	Frontal/Glabela/Orbicular/Nasal
Case 3	CBio	1	Sculptra	Lateral Face
	HA	1	^1^ restylane Volyme	Maxilla
	BTx	24 units Lower Third 36 units Upper Third	Dysport	Frontal/Glabela/Orbicular/Nasal/Platysma

CBio – Collagen biostimulator; HA – Hyaluronic acid; BTx – Botulinum toxin.

#### 2.4.1 Case 1

A 69-year-old female patient presented to the clinic with primary concerns of facial soft tissue alterations characterized by ‘facial melting’, along with the presence of static and dynamic wrinkles around the eyes and mouth area. Upon physical examination, a notable reduction in tissue thickness and loss of facial contours were observed.

**Fig. 1:**
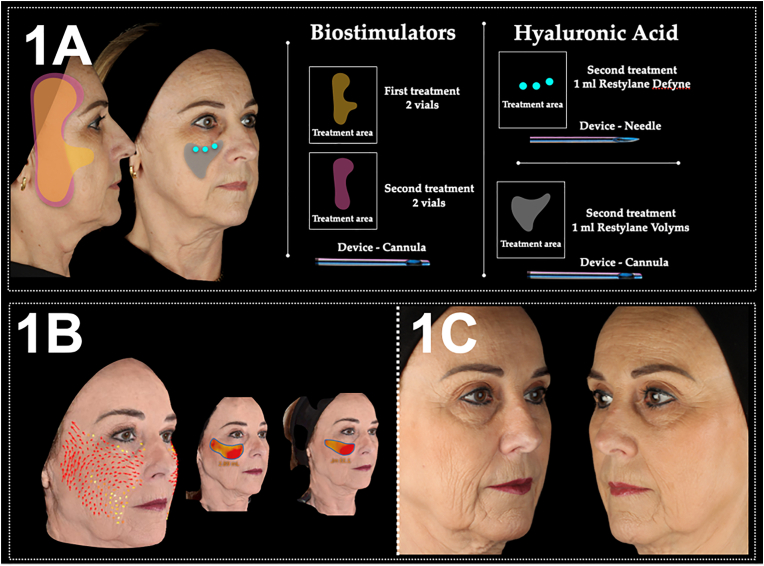
Firm, lyft and Relax 1A) approach protocol from case 1, and 1B) stereophotogrammetry and 1C) photographic results.

The initial treatment plan entailed the application of BTx to the upper and lower thirds of the face, along with the administration of 2 vials of PLLA to the temporal, preauricular, and mid-face regions. After 45 days, an additional vial of PLLA was utilized on the lateral face. Additionally, 1 ml of Restylane Defyne and 2 ml of Restylane Volyme were injected as detailed in Fig. 1A.

A noticeable skin quality improvement was observed 180 days after the first treatment. Additionally, cranial skin displacement and volume gain of 2.89 and 2.33 mL in the left and right midfacial region were obtained (Fig. 1B and C). No adverse events were reported by the patient.

#### 2.4.2 Case 2

A 35-year-old female patient presented with the primary concern of asymmetry in the lower third of the face. Photographic evaluation revealed a significant discrepancy between the right and left sides of the face, while physical examination indicated slight skin laxity. Additionally, atrophy in the temporal region resulted in a negative enhancement of the zygomatic arch and disproportion between the middle and lower thirds of the face.

**Fig. 2:**
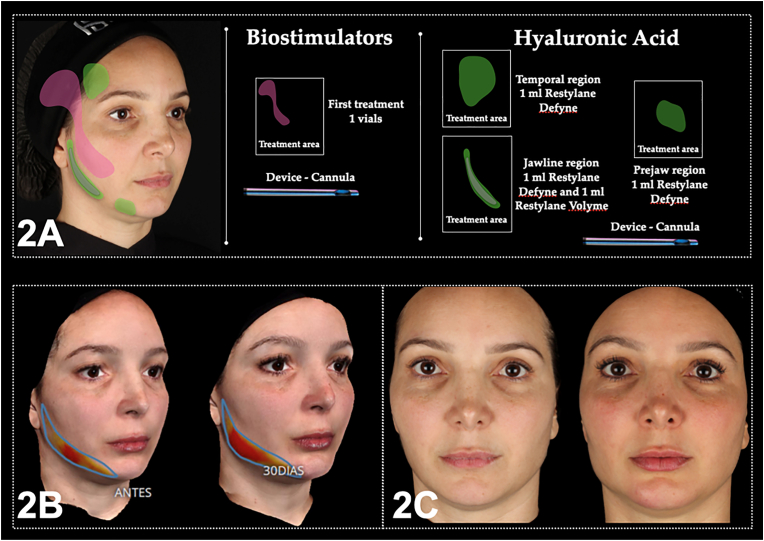
Firm, lyft and Relax 2A) approach protocol from case 2, and 2B) stereophotogrammetry and 2C) photographic results.

BTx was applied to the upper third of the face. Then, CBio was administered in the posterior temporal region and the submalar region using a 22G cannula (Biometik, Santa Catarina, Brazil). Importantly, injections of HA were exclusively carried out on the right jaw, combining 1 ml of Restylane Defyne and 1 ml of Restylane Volyme in the subcutaneous plane using a 22G cannula. In the temporal region, 0.5 ml of Restylane Volyme was applied per hemiface subcutaneously in the temporal fossa (Fig. 2A). Fig. 2B and C illustrate a notable improvement in both the temporal region and the right jaw. This progress is further supported by stereophotogrammetry, confirming the positive outcomes of the treatment in these specific areas. No adverse events were reported.

#### 2.4.3 Cases 3 and 4

Two female patients, both 39 years old, presented with the primary complaint of infraorbital hollows. Evaluation, including photographic and physical exams, revealed a significant volume loss in the mid-face region and a slight decrease in skin firmness and tissue support.

**Fig. 3:**
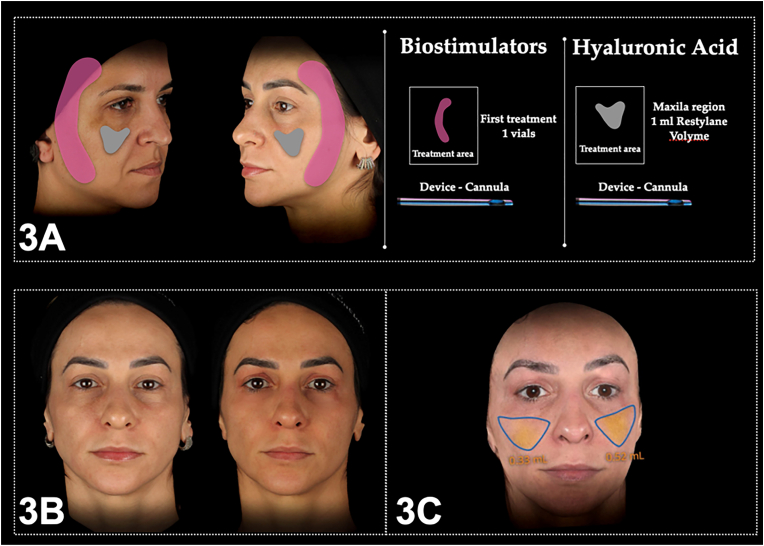
Firm, lyft and Relax 3A) approach protocol from case 3 and 4, and 3B) stereophotogrammetry and 3C) photographic results.

Both patients underwent an integrated approach combining BTx, CBio, and HA in a single session. The initial step involved applying BTx to the lower third of the face to induce tissue displacement, thereby enhancing volume and improving the appearance of the middle third and infraorbital region. Subsequently, CBio was administered to the lateral area of the face using a PLLA vial in the subcutaneous plane. Finally, injections of 0.5 ml of Restylane Volyme into the malar region were carried out subcutaneously using a 22G cannula (Fig. 3A). Fig. 3B demonstrate a clear improvement in the infraorbital region, while Fig. 3C shows 0.33 and 0.52 mL of volume gain in the left and right midfacial region respectively, addressing the primary concerns of both patients. These notable results underscore the success of the treatment in meeting the specific expectations and needs of both individuals. No adverse events were reported by the patients.

## 3 Discussion

Due to its effective results and straightforward application, the Firm & Lyft protocol^7^ has become a well-established method utilized by both experienced and novice injectors alike. Originally comprising only two types of HA and CBio (PLLA-Sculptra) in the preauricular area, this technique has evolved to incorporate CBio, HA, and BTx in a structured manner, thereby enhancing treatment customization to suit each patient's unique facial structure.

The results observed in the patients treated with the FL&R approach highlight the effectiveness of the combined use of BTx, HA, and CBio in addressing a wide range of aesthetic concerns. Significant improvements in facial appearance were noted, including increased tissue volume and repositioning, particularly in areas such as the midface and infraorbital region. These positive changes were confirmed by stereophotogrammetry and underscore the ability of FL&R to achieve comprehensive rejuvenation by effectively addressing both dynamic and static facial concerns, offering a tailored solution for each patient's specific needs.

While traditional facial rejuvenation methods, such as the isolated use of BTx or HA, have shown effective results in specific cases, the FL&R technique stands out for its ability to personalize treatment based on the individual needs of each patient. Rather than following a standardized approach, the FL&R strategy allows the clinician to focus on dynamic and static areas separately, addressing them individually to maximize results and preserve the natural movement of the face. Moreover, the combination of different CBio and HA, tailored according to tissue thickness and firmness, makes the FL&R approach versatile and adaptable, providing more precise solutions for a wide range of patients.

BTx should be administered before other procedures to maximize precision and minimize diffusion to adjacent muscles, particularly due to potential post-treatment edema. Tissue repositioning using BTx has been extensively demonstrated.^4^ Studies have shown that BTx injections in the mandibular region can lead to significant tissue repositioning and improvement of midfacial fullness.^16^ Furthermore, recent research by Germani and colleagues has shown improvement in infraorbital hollows following BTx injections into the platysma.^3^ Therefore, initiating most rejuvenation treatments with neuromodulators appears reasonable, as less product may be required in subsequent approaches due to this initial tissue repositioning.

Following neuromodulator administration, CBio may be utilized to enhance skin quality and complement soft tissue repositioning. CBio products are selected to stimulate collagen production, thereby improving skin quality^17^ and potentially promoting subtle volumization and tissue displacement over the long term.^18^ The selection of specific points and application areas is personalized, considering individual aesthetic goals and results from each patient assessment. In the FL&R strategy, CBio is distributed beyond the preauricular region, extending into the anterior and posterior temple, as well as the medial and submalar face, as documented in previous studies.^18^^,^^19^

Finally, HA applications aim to restructure or volumize facial regions where support or tissue volume has been lost.^1^ The selection of the appropriate HA product and injection plane depends on three main criteria: the degree of mobility of the area (static vs. dynamic areas), the need for tissue support or volume (projection vs. volumization), and tissue thickness. Clinicians must have a thorough understanding of the physicochemical characteristics and rheology of HA products to match the correct HA gel with the desired objectives.

No significant complications or adverse events were observed following the FL&R treatments in any of the cases. However, it is important to emphasize that the absence of adverse events in this small sample does not necessarily indicate that the FL&R approach is free from risks. Larger studies involving more diverse patient populations are required to thoroughly evaluate the safety profile and identify any potential long-term side effects.

The aim of this work was not to propose a standardized technique, but rather to develop a rejuvenation strategy utilizing the principles of facial biomechanics to address facial aging.^4^^,^^9^^,^^12^ In doing so, the adaptability of FL&R can benefit patients with diverse facial characteristics and concerns, particularly those with moderate signs of aging, such as volume loss and tissue laxity, as injectors have greater flexibility and discretion in selecting products. However, there remains a scarcity of studies demonstrating conclusive results from this combined approach, highlighting the need for further research to validate the findings presented and incorporating patient-reported outcomes, such as Likert scales or satisfaction assessments, to provide valuable objective data to further substantiate the efficacy of this technique.

## 4 Conclusion

The FL&R approach, combining BTx, HA, and CBio, has demonstrated effectiveness and versatility in facial rejuvenation. The case studies presented in this work underscore its efficacy in enhancing facial appearance and elevating patient satisfaction by addressing a range of aesthetic concerns with a personalized approach. This technique underscores the significance of customization in non-surgical cosmetic treatments, adapting to the unique needs of each patient to achieve optimal outcomes. However, further research is warranted to conclusively validate the efficacy of this combined approach.

## Regulatory statement

Patients provided informed consent for the publication of their photograph and for the use of their data in research.

## Ethical clearance

Kaiser Hospital Research Ethics Committee under protocol number CAAE - 75193923.8.0000.0281.

## Funding

This manuscript received no funding

## Declaration of competing interest

M.G. and D.D. are speakers from Galderma Brasil. K.V. is speaker from Galderma Chile. Other authors declare no conflict of interests.
